# Increased CMV IgG Antibody Titer is Associated with Non-AIDS Events among Virologically Suppressed HIV-Positive Persons

**DOI:** 10.20411/pai.v4i1.255

**Published:** 2019-02-14

**Authors:** Aimee C. Hodowanec, Nell S. Lurain, Supriya Krishnan, Ronald J. Bosch, Alan L. Landay

**Affiliations:** 1 Division of Infectious Diseases; Rush University Medical Center; Chicago Illinois; 2 Department of Immunology/Microbiology; Rush University Medical Center; Chicago Illinois; 3 Center for Biostatistics in AIDS Research; Harvard TH Chan School of Public Health; Boston Massachusetts

**Keywords:** HIV, cytomegalovirus (CMV), immunity, inflammation, malignancy, cardiovascular events

## Abstract

**Background:**

Among HIV-positive individuals, increased levels of inflammation and immune activation persist even in the setting of effective antiretroviral therapy (ART) and are associated with greater rates of non-AIDS events. The etiology of this persistent inflammation is incompletely understood.

**Methods:**

Using a well-characterized cohort of 322 HIV-infected individuals on suppressive ART, we conducted a case-control study. Cytomegalovirus (CMV) immunoglobulin G (IgG) levels, plasma biomarkers, and T-cell phenotypes were measured/characterized from samples collected 1 year after ART initiation. Conditional logistic regression for matched case-control studies analyzed the associations of year 1 CMV-specific IgG level with the subsequent occurrence of any non-AIDS event. Correlations between continuous CMV IgG antibody levels and soluble and cellular markers were assessed.

**Results:**

We found that higher levels of CMV IgG were associated with increased risk of non-AIDS events (OR = 1.58 per IQR [95% CI: 1.12, 2.24], *P* = 0.01) and with elevated soluble and cellular markers of inflammation.

**Conclusions:**

The magnitude of the host immune response to CMV may play a role in the persistent inflammation and resultant morbid events observed in the HIV-positive population.

## BACKGROUND

Untreated HIV infection is characterized by ongoing inflammation and immune activation [1]. Despite suppressive antiretroviral therapy (ART), many HIV-infected individuals continue to have increased inflammation compared to uninfected controls. Soluble markers of inflammation, such as interleukin (IL)-6, soluble tumor necrosis factor (TNF)-alpha receptor I and II, soluble CD14 and soluble CD163 and cellular markers of immune activation, such as HLA-DR/CD38 co-expressing T cells remain elevated in these individuals and are associated with morbid AIDS and non-AIDS events, including death [1-4].

The etiologies of these persistently elevated levels of inflammation and immune activation remain unclear, but are likely complex and variable across individuals. The direct effects of HIV, persistent HIV-related defects in the gut mucosal barrier with exposure to microbial products, reactivation of latent infections (eg, herpes viruses), chronic silent co-infections (eg, HCV) and persistent immune dysregulation are all potential drivers of ongoing inflammation and immune activation [1, 2].

With aging, host immune response is increasingly dominated by cytomegalovirus (CMV)-specific responses [5]. This disproportionate CMV-specific response might contribute to immune senescence and might be associated with inflammation. The presence of CMV infection has been associated with a variety of aging-related diseases in both HIV-positive and negative populations [6-9]. Among a large cohort of immunocompetent adults, higher anti-CMV immunoglobulin G (IgG) antibody levels were found to be associated with an increased incidence of ischemic heart disease and increased all-cause mortality [6, 7]. Similarly, one study on HIV-positive women found that higher anti-CMV IgG levels were associated with subclinical carotid artery disease, as diagnosed by carotid artery ultrasound in a subset of virologically suppressed participants [8]. Another study found that CMV seropositivity was associated with a significantly increased risk for severe non-AIDS events, in particular cardiovascular and cerebrovascular diseases [9]. It is not known whether these associations are causal in nature. Further, if there is a causal association between CMV and inflammatory conditions, it is not known whether this is due to a direct effect of CMV or if it is secondary to the inflammation associated with CMV-specific host immune responses. Further studies exploring the relationship between CMV and inflammatory conditions are needed, particularly among HIV-positive individuals.

In a previously reported case-control study within the AIDS Clinical Trials Group (ACTG) Longitudinal Linked Randomized Trials (ALLRT) cohort, soluble markers of inflammation (IL-6, sTNFR-I/II) and coagulation (D-dimer) measured 1 year after ART initiation were associated with subsequent non-AIDS events, but T-cell activation was not [10]. In this current study, we utilize the same cohort and soluble inflammatory marker and T-cell activation data to determine whether the magnitude of CMV-specific antibody response might be associated with the occurrence of non-AIDS events.

## METHODS

Participants from the ALLRT cohort were selected for this case-control study. Institutional Review Board (IRB) approval for ALLRT was obtained by each ACTG site. All participants provided written informed consent. As previously described by Tenorio *et al*., participants were ART-naive when enrolled into an ACTG study, had plasma HIV-1 RNA < 400 copies/mL at week 48 of ART, and maintained a plasma HIV-1 RNA < 400 copies/mL at all subsequent time-points (isolated values > 400 copies/ml were allowed.) Cases were defined as participants who developed a non-accidental, non-AIDS-related death, myocardial infarction (MI), stroke, or non-AIDS-defining malignancy or serious bacterial infection. One to three controls were identified for each case (median of two controls per case). Controls had an event-free follow-up time greater than the case and were matched for age, sex, pre-ART CD4+ T-cells (within ±50 cells/mm^3^), ART regimen at week 48 (whether protease-inhibitor containing or not, and whether abacavir-containing or not), and parent study [10]. Of the 458 subjects in the previously reported case-control study, 322 subjects had specimens available from the 1 year post-ART initiation timepoint and were included in the current study.

### Laboratory Analysis

All ALLRT participants had plasma, serum, and PBMCs collected, frozen, and stored at each study visit. For this study, specimens collected 1 year (48–64 weeks) after ART initiation were utilized. Quantitative CMV-specific IgG levels were measured using ELISA (GenWay Biotech Inc., San Diego, CA). Additionally, quantitative Epstein-Barr virus (EBV)-specific IgG levels were measured using ELISA (GenWay Biotech) to differentiate between a generalized increase in IgG levels and a CMV-specific increase in IgG levels. Year 1 plasma biomarker measurement (IL-6, sCD14, interferon-γ-inducible protein [IP-10], sTNFR-1, sTNFR-2, and D-dimer), T-cell phenotype characterization (immune activation [CD38+/HLA DR+ or PD1+], and immune senescence [CD57+/CD28-]) were available as part of the previously published study [10] and these results were included in our analysis.

### Statistical Analysis

It was anticipated that cases would have higher CMV IgG levels than controls. Conditional logistic regression for matched case-control studies analyzed the associations of year 1 CMV-specific IgG level (as a continuous variable) with subsequent occurrence of any non-AIDS event during follow-up. Of note, participants with a CMV IgG level < 1.2 IU/mL (CMV seronegative) were analyzed as CMV IgG = 1.2 IU/mL. Effects were quantified in terms of odds ratio (OR) per one interquartile range (IQR). Further analyses adjusted for year 1 CD4 count; smoking status; waist circumference; waist-hip ratio; and plasma levels of IL-6, IP-10, D-dimer, sCD14, sTNFR-1, and sTNFR-2. Additionally, CMV IgG antibody levels were classified into quartiles, and the OR was estimated for each of the top three quartiles relative to the lowest quartile. Subgroup analyses were performed by type of non-AIDS event, evaluating CMV IgG levels as a continuous variable. No adjustments were made for multiple comparisons. Correlations between continuous CMV IgG antibody levels and soluble and cellular markers were assessed using Spearman correlations (and 95% CI).

## RESULTS

Baseline characteristics of the 322 participants (107 cases and 215 controls) are presented in Table 1. Three-hundred and two (94%) participants were CMV seropositive at year 1. Cases and controls had similar age, pre-ART CD4 (matching variables), pre-ART HIV RNA, and year 1 HIV RNA. Among the 107 case subjects, the median (Q1-Q3) time from ART initiation to non-AIDS event was 2.9 (1.6–4.5) years.

**Table 1.** Demographic Characteristics of Cases and Controls

Variable	Total (N = 322)	Controls (N = 215)	Cases (N = 107)
Age, years; mean (SD)	45 (9)	44 (9)	46 (10)
Male sex	273 (85%)	183 (85%)	90 (84%)
Race/ethnicity			
White non-Hispanic	160 (50%)	104 (48%)	56 (52%)
Black non-Hispanic	98 (30%)	60 (28%)	38 (36%)
Hispanic (regardless of race)	58 (18%)	46 (21%)	12 (11%)
Other	6 (2%)	5 (2%)	1 (1%)
Pre-ART CD4, cells/mm^3^; mean (SD)	242 (174)	241 (172)	244 (177)
Year 1 CD4, cells/mm^3^; mean (SD)	420 (213)	431 (214)	399 (209)
Pre-ART HIV-1 RNA, log10 copy/mL; mean (SD)	4.8 (0.7)	4.9 (0.8)	4.7 (0.7)
Year 1 HIV-1 RNA, <50 copy/mL	287 (89%)	192 (89%)	95 (89%)
Year 1 CMV IgG, IU/mL			
Mean (SD)	35 (17)	33 (17)	38 (15)
Median (Q1, Q3)	37 (25, 47)	35 (22, 45)	39 (30, 49)
Year 1 waist circumference, cm; mean (SD)^*^	93 (13)	92 (13)	93 (13)
Year 1 waist-hip ratio; mean (SD)^**^	0.9 (0.1)	0.9 (0.1)	0.9 (0.1)
History of smoking	203 (63%)	122 (57%)	81 (76%)
Year 1 # cigarettes/day; mean (SD)	6 (10)	5 (9)	9 (11)

* Missing data for 25 participants

** Missing data for 26 participants

Results reported as n (%) unless otherwise noted

Abbreviations: ARV, antiretrovirals; CMV, cytomegalovirus; IgG, immunoglobulin G; SD, standard deviation

Comparison of the ORs for non-AIDS events by CMV IgG quartile revealed that, relative to the lowest CMV IgG quartile, the risk of non-AIDS events was higher for the remaining three quartiles. The ORs reached statistical significance for the fourth and third quartiles (OR = 2.4 [95% CI: 1.2, 5.0] and 2.2 [95% CI: 1.1, 4.4], respectively; *P* = 0.02 for both) but not the second quartile (OR = 1.6 [95% CI: 0.8, 3.3]; *P* = 0.16). Adjusting for year 1 CD4+, T-cell count did not significantly impact these associations. As previously noted, controls were matched for pre-ART CD4+ T-cell count; therefore no further adjustment for baseline CD4+ T-cell count was performed. Analysis of continuous CMV IgG levels and risk of non-AIDS events also showed a significant association (OR = 1.58 [95% CI: 1.12, 2.24], *P* = 0.01) that was maintained after adjusting for CD4 count (Table 2). Results were similar in a sensitivity analysis excluding CMV seronegative subjects (OR = 1.82, 95% CI 1.18, 2.80; *P* = 0.01). The significant association of continuous CMV IgG levels with non-AIDS events was also maintained when adjusted for inflammatory markers, cigarette use, waist circumference, and waist-hip ratio (data not presented). However, when specific non-AIDS events were analyzed individually (cardiovascular/cerebrovascular events, malignancy, and death), only malignancy was significantly associated with increased CMV IgG levels (Table 2). (See Supplementary Table 1 for a listing of the specific reported malignancies.) There were no significant associations between EBV IgG levels and combined non-AIDS events (unadjusted OR 1.24 [95% CI: 0.88, 1.75], *P* = 0.22) or between EBV and cardiovascular/cerebrovascular events (OR 0.74 [95% CI: 0.40, 1.36]) and malignancy (OR 1.65 [95% CI: 0.92, 2.94]) when analyzed individually. However, there was an association between EBV IgG level and death (OR 4.74 [95% CI: 1.11, 20.23], *P* = 0.036).

**Table 2.** Association between Year 1 CMV IgG Level and Non-AIDS Events

Event	Sample Size Case : Control	OR (95% CI)	*P*-value
Any Event	107^±^ : 215		
Unadjusted*		1.58 (1.12, 2.24)	0.01
Adjusted for year 1 CD4**		1.54 (1.08, 2.18)	0.02
MI/CVA	33 : 70		
Unadjusted*		1.36 (0.76, 2.44)	0.3
Adjusted for year 1 CD4**		1.36 (0.75, 2.46)	0.3
Malignancy	38 : 79		
Unadjusted*		1.99 (1.07, 3.70)	0.03
Adjusted for year 1 CD4**		2.07 (1.08, 3.94)	0.03
Death^#^	16 : 31		
Unadjusted*		0.86 (0.42, 1.76)	0.7
Adjusted for year 1 CD4**		0.77 (0.35, 1.71)	0.5

Abbreviations: CI, confidence interval; CMV, cytomegalovirus; CVA, cerebrovascular accident; IgG, immunoglobulin G; MI, myocardial infarction; OR, odds ratio per interquartile range of CMV IgG. Note that the CMV IQR was obtained by pooling cases and controls.

^±^ In addition to the MI/CVA, malignancy, and death non-AIDS events reported in this table, there were 26 serious bacterial infection non-AIDS events in case subjects. These non-AIDS events were not mutually exclusive as subjects may have had a bacterial infection, malignancy, or MI/CVA that resulted in death.

* From conditional logistic regression analysis which incorporates the case-control matching factors: age, sex, pre-ART CD4+ T-cell count, ART regimen and parent study.

** Further adjusting for year 1 CD4+ T-cell count.

^#^ The causes of death among these 16 case subject deaths were MI, malignancy, and serious bacterial infection (2 subjects each); and non-accidental death (n = 10, including chronic renal failure, end-stage liver disease, multi-system failure and congestive heart failure).

Spearman correlations were performed to assess the relationship between CMV IgG levels and biomarkers and cellular phenotypes. These analyses were performed among the controls to be most representative of the virally suppressed population of interest. CMV IgG antibody levels positively correlated with IL-6, sTNF-1, sTNF-2, CD4+ %PD-1+, CD4+ %28-57+, and CD4+ %DR+38+ (Table 3), and also with EBV IgG. After adjusting for CD4 count, the same associations were identified, with the exception of CD4+ %DR+38+ cells no longer being associated with CMV IgG level.

**Table 3.** Correlations between CMV IgG Level and Soluble and Cellular Immune Markers at Year 1 Among Controls

Variable	Unadjusted Analysis		Adjusted for CD4	
	Correlation (95% CI)	*P*-value	Correlation (95% CI)	*P*-value
CD4	-0.18 (-0.30 – -0.04)	0.009	-	-
CD8	0.14 (0.01 – 0.27)	0.04	-	-
CD4:CD8 ratio	-0.26 (-0.38 – -0.13)	< 0.001	-	-
IL-6	0.17 (0.04 – 0.30)	0.01	0.16 (0.02 – 0.29)	0.02
IP-10	0.08 (-0.06 – 0.21)	0.25	0.06 (-0.08 – 0.19)	0.40
D-dimer	0.05 (-0.08 – 0.19)	0.43	0.05 (-0.08 – 0.18)	0.46
sCD14	0.03 (-0.11 – 0.16)	0.69	0.01 (-0.13 – 0.14)	0.89
sTNFR-1	0.25 (0.12 – 0.37)	< 0.001	0.22 (0.09 – 0.35)	0.001
sTNFR-2	0.26 (0.13 – 0.38)	< 0.001	0.24 (0.11 – 0.36)	< 0.001
CD4+ %PD-1+	0.22 (0.08 – 0.35)	0.002	0.15 (0.01 – 0.28)	0.04
CD4+ %28-57+	0.20 (0.06 – 0.33)	0.005	0.19 (0.05 – 0.32)	0.008
CD4+ %DR+38+	0.15 (0.01 – 0.28)	0.04	0.07 (-0.07 – 0.21)	0.32
CD8+ %PD-1+	-0.12 (-0.25 – 0.02)	0.11	-0.12 (-0.26 – 0.02)	0.09
CD8+ %28-57+	0.10 (-0.04 – 0.23)	0.17	0.07 (-0.07 – 0.20)	0.35
CD8+ DR+38+	0.10 (-0.04 – 0.23)	0.18	0.05 (-0.09 – 0.19)	0.47
EBV IgG	0.22 (0.08 – 0.34)	0.001	0.18 (0.05 – 0.31)	0.008

Abbreviations: CI, confidence interval; EBV, Epstein-Barr Virus; IgG, immunoglobulin G; IL-6, interleukin 6; IP-10, interferon γ–inducible protein 10; sCD14, soluble CD14; sTNFR, soluble tumor necrosis factor receptor.

## DISCUSSION

CMV seroprevalence is high among HIV-positive individuals and CMV infection has been associated with numerous inflammatory conditions [6-9]. However, whether CMV is an active participant or an innocent bystander in the occurrence of these inflammatory processes among the HIV/CMV-coinfected population remains controversial. Among virologically suppressed HIV-positive individuals, we found a significant association between a higher CMV IgG level and the occurrence of non-AIDS events overall. This finding is consistent with the findings of Lichtner *et al*., though the Lichtner study compared CMV-seropositive participants to CMV-seronegative participants, rather than assessing quantitative CMV IgG levels [9]. Of note, among participants included in our study, the CMV IgG levels at Year 1, though higher in cases than in controls, show substantial overlap in their distributions. This suggests that CMV IgG levels may not be reliable as a biomarker to predict who will or will not experience a non-AIDS event. Rather, CMV IgG levels may be more informative as a biomarker evaluated at a population level.

Further characterization of the relationship between CMV IgG levels and non-AIDS events is impeded by an incomplete understanding of the biological basis for increased CMV IgG levels. It has been suggested that high CMV IgG levels may be caused by frequent CMV reactivations [6]. Alternatively, CMV reactivation could be a result of inadequate CMV-specific immunity [11]. Under this hypothesis, a high CMV IgG level would be associated with infrequent CMV reactivation.

Although the observed association between CMV IgG level and non-AIDS events does not confirm a causal relationship, the associations between CMV IgG level and several markers of inflammation and immune activation allude to a potential mechanism for this association. In particular, we found that among control participants there was a weak but statistically significant positive association between s IL-6, sTNF1, sTNF2, CD4+ %PD-1+, CD4+ %28-57+, and CD4+ %DR+38+and CMV IgG levels. These same soluble markers of inflammation measured in this same cohort were also associated with increased risk of non-AIDS events at both 1 year post-ART initiation and at a timepoint immediately prior to the non-AIDS event. However, the cellular markers of immune activation were not [10]. These correlations suggest that chronic CMV infection may lead to increased inflammation, immune activation, and immune senescence. Chronic inflammation among patients with an increased humoral CMV-specific immune response may be a driver of non-AIDS events and malignancy in this population. Based on the findings from this cohort, the role of immune activation in the occurrence of non-AIDS events is less clear.

sCD14 has previously been identified as a predictor of mortality among HIV-positive persons [3]. Similarly, Tenorio *et al*. previously reported that, in our current cohort, sCD14 levels at a time-point immediately preceding the non-AIDS event, but only marginally at 1 year post-ART initiation, were positively associated with the risk of non-AIDS events [10]. Our study did not find an association between sCD14 and CMV IgG levels at one year post-ART initiation. Unfortunately, CMV IgG levels are not available for the pre-event timepoint for this cohort. These findings highlight the dynamic nature of these various biomarkers and suggest that monocyte activation may play a role in the inflammatory environment contributing to the occurrence of non-AIDS events.

Our finding that CMV IgG levels are associated with increased rates of malignancy among HIV-positive individuals has not previously been reported and the mechanism for this finding is unknown. To date, there is no clear evidence that CMV is directly oncogenic [12]. However, CMV DNA and antigens have been detected in breast cancer tissues and brain tumors [13, 14]. Additionally, it is possible that, through its immune modulating activities, CMV interferes with immune checkpoints that would otherwise slow or prevent cancer growth. Alternatively, as many of the same immunologic pathways are involved in both controlling viral infections and preventing malignancy, higher CMV IgG levels in cases with cancer may be a result of rather than a cause of immunologic derangements. The robustness of the observed association between CMV IgG levels and malignancy is uncertain. In the absence of another study confirming this association, our findings should be interpreted cautiously.

While EBV and CMV IgG levels were correlated (r = 0.22, adjusted r = 0.18), EBV IgG levels were not predictive of non-AIDS events, while CMV IgG significantly predicted both overall non-AIDS events and malignancies. This suggests that the increased risk of clinical events is in fact driven by a CMV immune response rather than by a generalized IgG response. However, a significant correlation between EBV IgG level and death was observed and cannot be explained. It may be useful for this to be evaluated in a separate cohort.

Our study has several limitations. First, it is a case-control study and hence has the potential for confounding. We addressed this through matching in the study design and by the consistency of the findings in adjusted models. Further, CMV IgG level and cellular and soluble markers of inflammation were measured at a single timepoint, not at the time of the non-AIDS event. Therefore, assessment of the immunologic and inflammatory milieu at the time of the event is not possible. Another limitation that this study shares with many studies exploring the impact of CMV-coinfection in HIV-positive patients is a limited number of CMV seronegative participants. This is due to the overall high seroprevalence of CMV among adults and the particularly high seroprevalence of CMV among certain subsets of HIV-positive patients. However, previous studies suggest that, in the overall population, CMV seropositivity, regardless of the magnitude of antibody response, is associated with an increased risk of cardiovascular disease [15]. Additionally, a large prospective, observational study of HIV-positive persons found an association between CMV seropositivity and risk of non-AIDS events. This association was most pronounced for cardiovascular/cerebrovascular events [9]. Lastly, data regarding the potential history of CMV disease and antiviral therapy usage among participants is not available for this cohort. Exposure of participants to antiviral agents with anti-CMV activity may impact CMV IgG levels as well as rates of non-AIDS events.

This study supports previous studies suggesting that there is an association between CMV infection and various inflammatory conditions among HIV-positive patients. To the best of our knowledge, this is the first study identifying a correlation between the magnitude of host humoral response towards CMV and malignancy among HIV-positive individuals. The cellular and soluble inflammatory marker data available for participants in this cohort provide additional insight regarding potential mechanisms for these relationships, and the role of CMV co-infection in treated HIV-infected individuals is an active area of research [16, 17]. Future investigations to probe the mechanism would be valuable, including replication of our main findings [18], measurement of IgG subclasses and other measures of B-cell activation [19], as well as directly measuring CMV replication [20] (which is currently planned for this same cohort). Prospective interventional studies utilizing anti-CMV antiviral therapy may ultimately be needed to determine whether CMV plays a direct role in the occurrence of non-AIDS events.

## CURRENT AFFILIATIONS

Aimee Hodowanec's affiliation has changed. She is now a medical officer at the US Food and Drug Administration, Center for Drug Evaluation and Research, Office of New Drugs, Division of Antiviral Products (10903 New Hampshire Avenue, Silver Spring, MD 20993). However, all of the work for this project was completed while she was on the faculty at Rush University Medical Center, Division of Infectious Diseases.

## SUPPLEMENTARY MATERIALS

**Supplementary Table 1.** Specific Malignancies Reported Among Case Subjects

Malignancy (Reported Preferred Term)	Number of Cases
Anal cancer	3
Basal cell carcinoma	7
Breast cancer female	1
Colon cancer	1
Gastric cancer	1
Hodgkin's disease	6
Hypopharyngeal cancer	1
Large cell carcinoma of the respiratory tract stage unspecified	1
Lung adenocarcinoma	1
Lung cancer metastatic	1
Lung neoplasm	1
Lung neoplasm malignant	1
Metastatic neoplasm	1
Neoplasm malignant	2
Pharyngeal cancer stage unspecified	1
Prostate cancer	3
Rectal cancer	2
Small cell lung cancer metastatic	1
Small cell lung cancer stage unspecified	1
Testicular neoplasm	1
Thyroid cancer	1
